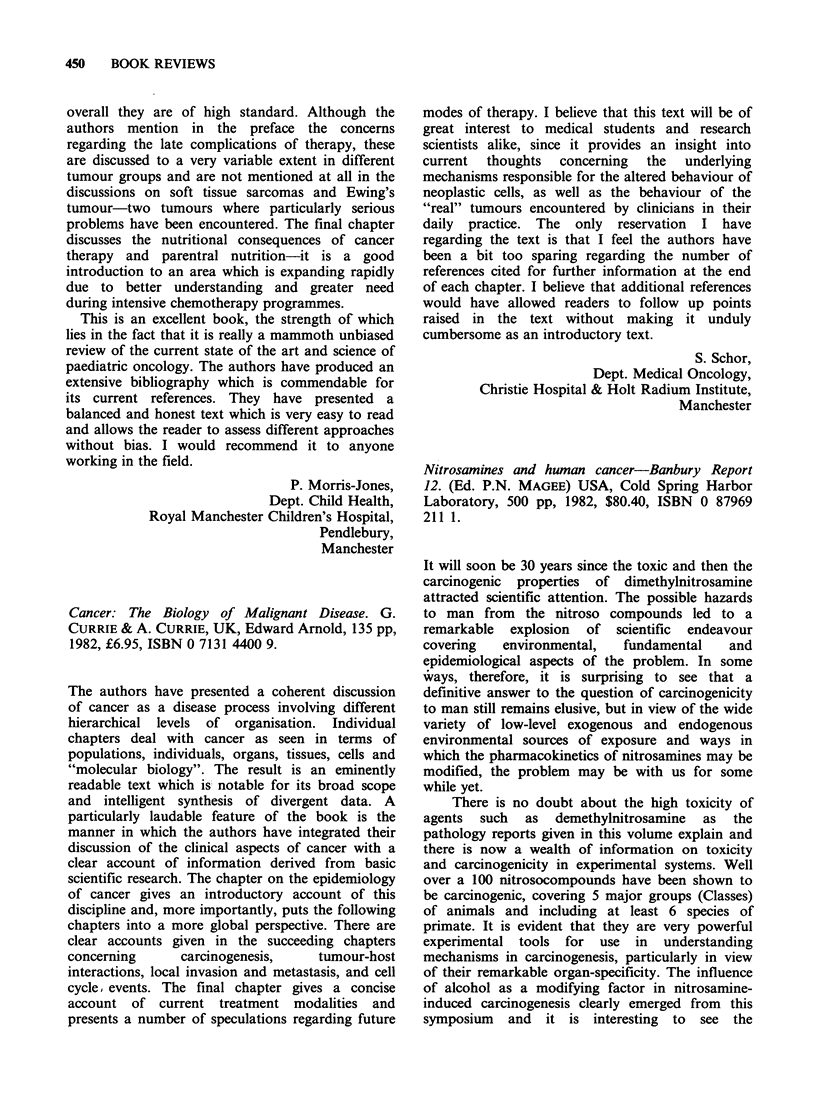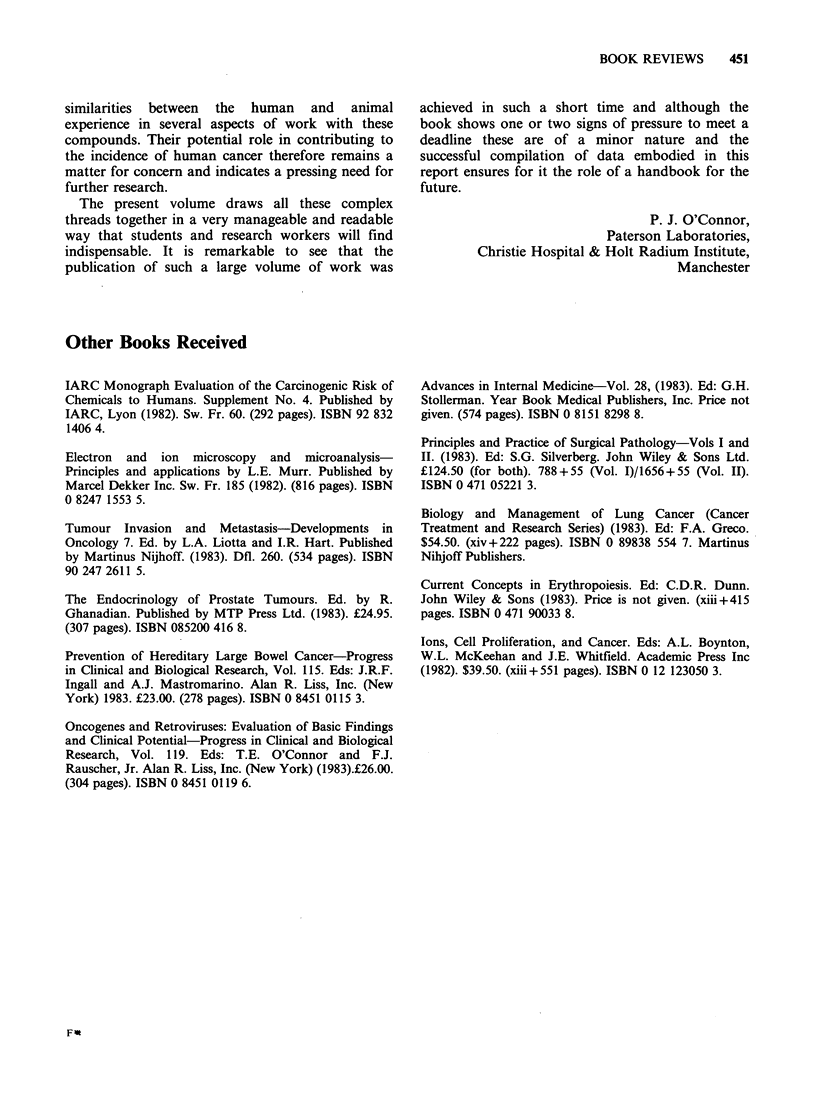# Nitrosamines and human cancer—Banbury Report 12

**Published:** 1983-09

**Authors:** P.J. O'Connor


Christie Hospital & Holt Radium Institute,

Manchester

*Nitrosamines and human cancer—Banbury Report 12.* (Ed. P.N. MAGEE) USA, Cold Spring Harbor Laboratory, 500 pp, 1982, $80.40, ISBN 0 87969 211 1.

It will soon be 30 years since the toxic and then the carcinogenic properties of dimethylnitrosamine attracted scientific attention. The possible hazards to man from the nitroso compounds led to a remarkable explosion of scientific endeavour covering environmental, fundamental and epidemiological aspects of the problem. In some ways, therefore, it is surprising to see that a definitive answer to the question of carcinogenicity to man still remains elusive, but in view of the wide variety of low-level exogenous and endogenous environmental sources of exposure and ways in which the pharmacokinetics of nitrosamines may be modified, the problem may be with us for some while yet.

There is no doubt about the high toxicity of agents such as demethylnitrosamine as the pathology reports given in this volume explain and there is now a wealth of information on toxicity and carcinogenicity in experimental systems. Well over a 100 nitrosocompounds have been shown to be carcinogenic, covering 5 major groups (Classes) of animals and including at least 6 species of primate. It is evident that they are very powerful experimental tools for use in understanding mechanisms in carcinogenesis, particularly in view of their remarkable organ-specificity. The influence of alcohol as a modifying factor in nitrosamine-induced carcinogenesis clearly emerged from this symposium and it is interesting to see the similarities between the human and animal experience in several aspects of work with these compounds. Their potential role in contributing to the incidence of human cancer therefore remains a matter for concern and indicates a pressing need for further research.

The present volume draws all these complex threads together in a very manageable and readable way that students and research workers will find indispensable. It is remarkable to see that the publication of such a large volume of work was achieved in such a short time and although the book shows one or two signs of pressure to meet a deadline these are of a minor nature and the successful compilation of data embodied in this report ensures for it the role of a handbook for the future.

P. J. O'Connor,
Paterson Laboratories,
Christie Hospital & Holt Radium Institute,
Manchester